# Cholesterol-Enriched Hybrid Lipid Bilayer Formation on Inverse Phosphocholine Lipid-Functionalized Titanium Oxide Surfaces

**DOI:** 10.3390/biomimetics8080588

**Published:** 2023-12-04

**Authors:** Tun Naw Sut, Joshua A. Jackman, Nam-Joon Cho

**Affiliations:** 1 School of Materials Science and Engineering, Nanyang Technological University, 50 Nanyang Avenue, Singapore 639798, Singapore; suttunnaw@skku.edu; 2 School of Chemical Engineering and Translational Nanobioscience Research Center, Sungkyunkwan University, Suwon 16419, Republic of Korea

**Keywords:** hybrid lipid bilayer, inverse phosphocholine, self-assembled monolayer, cholesterol, solvent exchange, vesicle fusion, quartz crystal microbalance-dissipation

## Abstract

Hybrid lipid bilayers (HLBs) are rugged biomimetic cell membrane interfaces that can form on inorganic surfaces and be designed to contain biologically important components like cholesterol. In general, HLBs are formed by depositing phospholipids on top of a hydrophobic self-assembled monolayer (SAM) composed of one-tail amphiphiles, while recent findings have shown that two-tail amphiphiles such as inverse phosphocholine (CP) lipids can have advantageous properties to promote zwitterionic HLB formation. Herein, we explored the feasibility of fabricating cholesterol-enriched HLBs on CP SAM-functionalized TiO_2_ surfaces with the solvent exchange and vesicle fusion methods. All stages of the HLB fabrication process were tracked by quartz crystal microbalance-dissipation (QCM-D) measurements and revealed important differences in fabrication outcome depending on the chosen method. With the solvent exchange method, it was possible to fabricate HLBs with well-controlled cholesterol fractions up to ~65 mol% in the upper leaflet as confirmed by a methyl-β-cyclodextrin (MβCD) extraction assay. In marked contrast, the vesicle fusion method was only effective at forming HLBs from precursor vesicles containing up to ~35 mol% cholesterol, but this performance was still superior to past results on hydrophilic SiO_2_. We discuss the contributing factors to the different efficiencies of the two methods as well as the general utility of two-tail CP SAMs as favorable interfaces to incorporate cholesterol into HLBs. Accordingly, our findings support that the solvent exchange method is a versatile tool to fabricate cholesterol-enriched HLBs on CP SAM-functionalized TiO_2_ surfaces.

## 1. Introduction

Lipid bilayer coatings on inorganic surfaces are widely referred to as supported lipid bilayers (SLBs) and are useful in various biotechnological applications such as biosensing and implant coatings [1,2]. First reported by Brian and McConnell, SLBs were originally designed as a biomimetic lipid membrane platform to interface with cells and have since been the subject of intense investigation to understand the fundamentals of phospholipid bilayers due to the possibility of characterizing SLBs at solid–liquid interfaces with a wide range of surface-sensitive measurement techniques [3,4]. SLBs have also proven useful as functional biointerfaces since they can inhibit nonspecific biofouling or promote specific biomolecular binding processes depending on the membrane composition [5,6,7,8]. This versatility has further led researchers to explore fabricating SLBs of diverse compositions on different inorganic surfaces, prompting the development of robust SLB fabrication protocols [9].

While various fabrication options exist, SLBs are typically formed by depositing solution-phase lipid assemblies (e.g., vesicles and bicelles) onto certain solid surface types (usually silica-based materials), followed by adsorption and spontaneous rupture processes that can trigger lipid reassembly and SLB formation [10,11]. In such cases, lipid–surface interactions involving the deposited lipid assemblies are noncovalent and need to be sufficiently strong for SLB formation to proceed. From an application perspective, however, since the only stabilizing factor is relatively weak noncovalent forces, the resulting SLBs are unstable in harsh environments due to chemical and physical cues like organic solvent and detergent treatments [12,13].

To address this limitation, hybrid lipid bilayers (HLBs) are another type of reinforced model membrane that expand on the SLB design concept and have more robust stability in harsh environments [14,15,16]. HLBs are formed by first covalently attaching the lower leaflet of amphiphilic lipid or lipid-like molecules to the solid surface and then depositing phospholipids on top of the exposed lower leaflet to form the upper leaflet via hydrophobic interactions. The covalently attached lower leaflet behaves like a self-assembled monolayer (SAM) and remains stable even after intentionally removing the noncovalently adsorbed upper leaflet (e.g., via organic solvent washing) [17]. The covalent attachment of the lower leaflet to the underlying solid support is the design element that makes HLBs mechanically rugged and ensures high stability over long periods for up to months, as has been reported [14]. At the same time, the upper leaflet of the HLB retains the properties of natural cell membranes such as self-assembled organization driven by noncovalent interactions and a degree of lateral lipid mobility [18,19]. Hence, HLBs are preferred to SLBs for applications that require the biomimetic lipid coating to be stable against environmental challenges and reusable even after multiple washing steps [20].

In addition to providing stability, HLBs expand the range of material surfaces on which biomimetic lipid membrane platforms can be fabricated. This capability is achieved by first forming a hydrophobic SAM coating upon which vesicle fusion can be triggered, whereas vesicle fusion on hydrophilic surfaces to form SLBs is typically limited to silica-based materials only [21,22]. Traditionally, for HLB fabrication, single-tail alkylthiols are used to prepare SAMs on metals (e.g., Au and Ag), whereas single-tail alkylsilanes are used on oxides (e.g., SiO_2_ and TiO_2_) [23]. Recently, the use of two-tail inverse phosphocholine (CP) lipids [24,25,26,27] to form SAMs on flat TiO_2_ surfaces based on phosphate chemistry (i.e., covalent P-O-Ti bond formation [28]) was also reported and enabled HLB formation across a well-defined set of ionic strength and pH conditions [19,29,30]. Compared to traditional silanization that requires organic solvent, high temperature, and/or a long incubation time (with special surface pretreatment in some cases), the CP lipid attachment scheme on TiO_2_ is simpler because it can readily occur in aqueous solution and at room temperature with shorter incubation time and no special surface pretreatment (other than straightforward oxygen plasma treatment) [31]. Therefore, CP-based SAM formation is particularly promising from a fabrication perspective (e.g., to prepare HLBs on biomedically relevant TiO_2_) and also advantageous from a biomimetic perspective since the attached CP lipids have two hydrocarbon chains (i.e., two tails) per molecule that resembles the structural arrangement of natural phospholipids.

So far, vesicle fusion driven by hydrophobic interactions has been the preferred deposition method to fabricate the upper leaflet of HLBs [32], the composition of which can be readily tuned by varying the lipid composition of precursor vesicles. In general, zwitterionic lipid vesicles can readily fuse and rupture on a SAM to form the upper leaflet [33] while there is also interest in incorporating more biologically relevant components such as sterols. Within the latter scope, recent studies have demonstrated the effect of cholesterol (Chol) on modulating the physicochemical properties, barrier functions, and mechanical stability of various biological membrane models [34,35,36,37]. In this context, HLBs have proven to be a particularly useful model to study cholesterol-related membrane properties since zwitterionic lipid vesicles containing up to ~40 mol% Chol can form the upper leaflet of HLBs on top of single-tail SAMs [20,38,39], whereas vesicles containing only up to ~20 mol% Chol can rupture to form SLBs on SiO_2_ surfaces [40,41]. In the two-chain CP SAM context, we have previously demonstrated that upper leaflet formation for HLBs on TiO_2_ can be achieved using zwitterionic lipid vesicles that contain a fraction of biotinylated lipids [31], but Chol incorporation into CP SAM-based HLBs has not been attempted. In addition to the vesicle fusion method, the solvent exchange method has excellent potential for Chol incorporation into the upper leaflet of CP SAM-based HLBs because the method can form SLBs on SiO_2_ with lipid mixtures containing up to around 63 mol% Chol [40,41]. Considering these factors, there is an outstanding need to evaluate the feasibility of fabricating cholesterol-enriched HLBs on TiO_2_ surfaces with two-tail CP SAMs and to further compare the efficiency of Chol incorporation using different fabrication methods. From a broader scope, it is also important to investigate the potential to fabricate HLBs on DOCP-based SAMs by using the solvent exchange method, which does not require vesicle preparation and would provide a more facile approach than the vesicle fusion method that has been the only one used so far.

**Figure 1:**
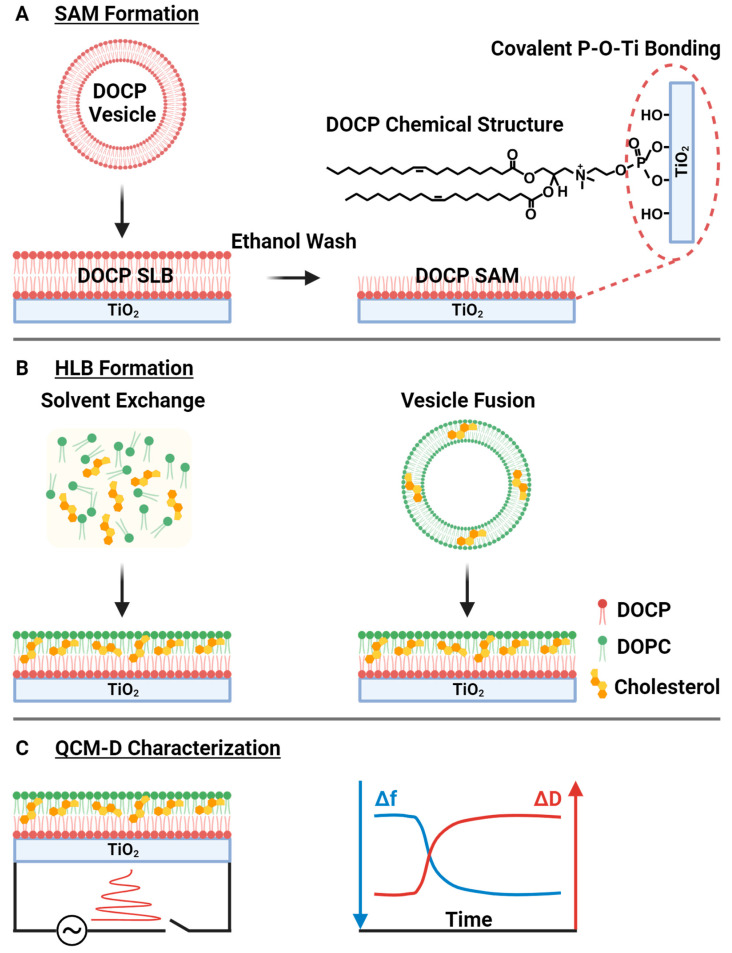
Experimental strategy for Chol-enriched HLB fabrication on CP SAM interfaces. (**A**) The CP SAM was first formed on TiO_2_ by CP lipid vesicle adsorption leading to CP SLB formation, followed by removal of the upper leaflet with ethanol washing. (**B**) The Chol-containing upper leaflet was then formed by the vesicle fusion or solvent exchange method to complete HLB fabrication. (**C**) HLB formation was characterized by the QCM-D technique that temporally tracks changes in the resonance frequency (Δf) and energy dissipation (ΔD) properties of oscillating TiO_2_-coated quartz crystals during the HLB formation process.

Thus, we systematically investigated the fabrication of Chol-enriched HLBs on CP SAM-functionalized TiO_2_ surfaces by comparing the vesicle fusion and solvent exchange methods (Figure 1). We first formed CP SAMs on TiO_2_ surfaces by adsorbing CP lipid vesicles followed by an ethanol washing step to leave only the covalently attached lower lipid leaflet. Then, we formed the upper lipid leaflet with Chol-containing lipid samples according to the vesicle fusion and solvent exchange methods. All stages of the fabrication process, including real-time adsorption kinetics and resulting adlayer properties, were characterized by the quartz crystal microbalance-dissipation (QCM-D) technique, which provides insight into the mass and viscoelastic properties of the adsorbate. A methyl-β-cyclodextrin (MβCD) extraction assay was also implemented to correlate HLB fabrication properties with resulting levels of cholesterol incorporation, allowing us to identify how the choice of fabrication method and input cholesterol fraction impacted the biomimetic character of the HLB platform.

## 2. Materials and Methods

Materials. Chloroform solutions of 2-((2,3-bis(oleoyloxy)propyl)dimethylammonio)ethyl hydrogen phosphate (DOCP), 1,2-dioleoyl-*sn*-glycero-3-phosphocholine (DOPC), and cholesterol (Chol) were obtained from Avanti Polar Lipids (Alabaster, AL, USA). Ethanol, isopropanol, and methyl-β-cyclodextrin (MβCD), along with other reagents, were purchased from Sigma-Aldrich (St. Louis, MO, USA). The Tris buffer (10 mM Tris, 150 mM NaCl, pH 7.5) was prepared with Milli-Q-treated water (MilliporeSigma, Burlington, MA, USA).

Vesicle Preparation. DOCP and DOPC lipid vesicles were prepared by the extrusion method, as previously described [42]. Briefly, chloroform solutions of DOCP and DOPC (with desired mol% Chol) were dried in a glass vial with nitrogen gas, and the resulting dry lipid films were desiccated in a vacuum chamber overnight to remove chloroform residues. For extrusion, the films were hydrated in Tris buffer to 5 mg/mL, and the lipid suspensions were passed through a polycarbonate filter with 50 nm diameter pores by using a mini-extruder device (Avanti Polar Lipids). Before experiments, the vesicles were diluted in equivalent Tris buffer to 0.1 mg/mL.

Quartz Crystal Microbalance-Dissipation (QCM-D). All stages of the HLB fabrication process were monitored by the QCM-D technique [43] using a Q-Sense E4 instrument (Biolin Scientific AB, Gothenburg, Sweden). The QCM-D crystals were sputter-coated with 50 nm thick TiO_2_ layers and used as supplied by the manufacturer (QSX310, Biolin Scientific AB). The primary phase of the TiO_2_ coating has been reported to be anatase [44], with a surface roughness of ~0.5 nm [45]. The crystals had a fundamental resonance frequency of 5 MHz and a mass sensitivity constant of 17.7 ng/cm^2^ per Hz. Before each experiment, the crystals were rinsed with water and ethanol, dried with nitrogen gas, and treated with oxygen plasma for 3 min in an Expanded Plasma Cleaner (PDC-002, Harrick Plasma, Ithaca, NY, USA). All solutions were injected into the measurement chambers at 50 µL/min using a peristaltic pump (Reglo Digital MS-4/6, Ismatec, Glattbrugg, Switzerland). The measurement data were collected at odd overtones (3rd–11th) using the Q-Soft401 software program (Biolin Scientific AB; version no. 2.5.28.732), and the normalized data at the 5th overtone are reported.

## 3. Results and Discussions

We employed the QCM-D technique to investigate HLB formation as a function of Chol fraction in the upper leaflet because it is a useful measurement approach to quantitatively characterize lipid adsorption kinetics and adlayer properties [46,47,48]. In particular, the QCM-D technique can track the changes in the frequency (Δf) and energy dissipation (ΔD) signals of an oscillating quartz crystal, which are related to the mass and viscoelastic properties of the adsorbed layer, respectively. Typically, Δf decreases when there is mass adsorption, and ΔD increases when the adsorbed mass is more viscoelastic, and vice versa. For rigid bilayers, the final Δf values range from −25 to −30 Hz depending on the lipid composition, and the ΔD values were around <1 × 10^−6^, which indicates rigid attachment [49]. The experimental conditions were fixed at pH 7.5, 150 mM NaCl salt concentration, and 25 °C temperature, which are generally representative, near-physiological conditions that are typical for model membrane fabrication, while the main varied parameter was the input Chol fraction across the two tested fabrication methods.

Compared to other surface-sensitive measurement options like atomic force microscopy (AFM) and surface plasmon resonance (SPR), we selected QCM-D as the main experimental technique because it has high kinetic resolution and its simultaneous measurement of Δf and ΔD signals permits detailed characterization of adsorbate properties, including distinguishing complete HLB formation from the presence of unruptured vesicles or other aggregate-type lipid assemblies that may form at the SAM interface. Such measurement capabilities have led to its wide usage for characterizing lipid adsorption processes (see also Refs. [46,50] for a detailed discussion of QCM-D merits). Whereas high-quality HLBs behave like non-viscoelastic films with low energy dissipation (hence, negligible ΔD shifts), unruptured lipid vesicles contain high amounts of hydrodynamically coupled solvent and behave like viscoelastic films with high energy dissipation (hence, large ΔD shifts). Since the Δf signal is proportional to the adsorbed mass for rigid films like HLBs, it was also possible to quantify the incorporated amount of Chol by MβCD assay. Thus, the SAM and HLB fabrication processes and resulting adlayer properties along with cholesterol incorporation level could all be readily measured and quantified by the QCM-D technique using well-established measurement principles.

### 3.1. DOCP SAM Formation

We selected two-tail DOCP molecules to form SAMs on TiO_2_ because they mimic the basic molecular properties of natural phospholipids and have also proven useful for robust HLB fabrication using vesicle fusion, whereas vesicles adsorbed but did not fully rupture on SAMs composed of one-tail phosphonic acid counterparts [31]. The difference in vesicle fusion efficiency to form the HLB on TiO_2_ was previously attributed to differences in SAM packing order [51] between one-tail vs. two-tail SAMs.

**Figure 2:**
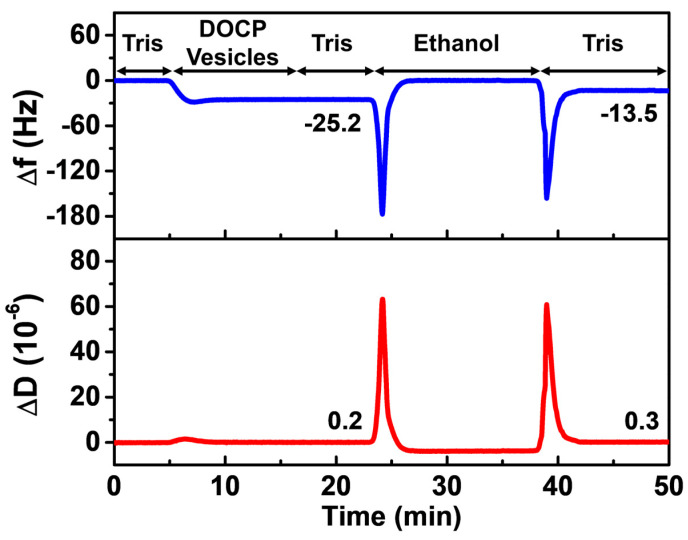
QCM-D monitoring of DOCP SAM formation on TiO_2_. DOCP SAM was formed by the following protocol: Tris buffer baseline, DOCP vesicle adsorption, Tris buffer wash, ethanol wash, and lastly Tris buffer exchange. The vesicle adsorption process led to DOCP SLB formation with Δf and ΔD shifts around −25 Hz and <1 × 10^−6^, respectively, while the ethanol washing and subsequent Tris buffer exchange steps led to a DOCP SAM with final Δf and ΔD shift values around −13 to −15 Hz and <1 × 10^−6^, respectively.

In the present case, we first formed a DOCP SAM on TiO_2_ with the following experimental protocol (Figure 2): Tris buffer baseline, DOCP lipid vesicle adsorption, Tris buffer washing, ethanol washing, and lastly Tris buffer exchange. After establishing the Tris buffer baseline, vesicle adsorption occurred with two-step interaction kinetics, i.e., adsorption until reaching a critical surface coverage followed by vesicle rupture. After Tris buffer washing following vesicle adsorption, the Δf and ΔD values reached around −25 Hz and <1 × 10^−6^, respectively, which are consistent with bilayer formation [49]. Then, the upper leaflet of the DOCP SLB was removed by ethanol rinsing and a subsequent Tris buffer exchange step that collectively resulted in SAM formation, i.e., a single layer of DOCP molecules organized on the TiO_2_ surface.

The introduction of ethanol to the DOCP SLB initially caused appreciable changes in Δf and ΔD signals due to the substantial differences in bulk viscosity and density between Tris buffer and ethanol, but these bulk effects on the measurement responses were only transient and fully reversible after re-exchanging back to Tris buffer solution in the next protocol step. The final Δf and ΔD values after the last Tris buffer exchange step were around −13 to −15 Hz and <1 × 10^−6^, respectively, indicating that the DOCP SAMs were attached rigidly in a manner consistent with typical SAM assemblies [46]. According to the Sauerbrey equation that converts Δf shifts into areal mass densities for rigid, non-viscoelastic adsorbates, the calculated mass is in the range of around 230 to 265 ng/cm^2^, which is about half the value of a complete and continuous PC-based SLB [46,52] and thus indicates complete coverage of the DOCP SAM.

### 3.2. Chol-Containing Upper Leaflet Formation

Next, we fabricated upper leaflets containing different molar ratios of zwitterionic DOPC phospholipid and Chol on top of DOCP SAMs in order to obtain Chol-enriched HLBs by using two different methods—solvent exchange and vesicle fusion. We prepared DOPC/Chol samples with varying Chol fractions between 0 and 65 mol% to empirically determine the compositional range of HLB fabrication utility using each method, while Chol fractions in different biological membrane types can vary but are usually within this range.

#### 3.2.1. Solvent Exchange

**Figure 3:**
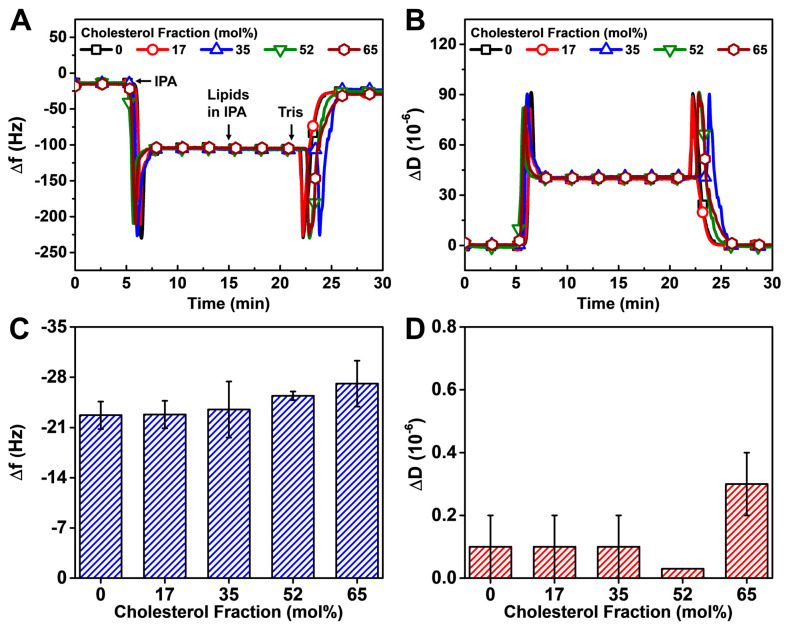
QCM-D monitoring of HLB formation with DOPC/Chol upper leaflets on DOCP SAMs by the solvent exchange method. The QCM-D kinetic profiles of (**A**) resonance frequency (Δf) and (**B**) energy dissipation (ΔD) shifts during the upper leaflet formation process that includes IPA exchange, injection of DOPC/Chol mixture in IPA, and exchange to Tris buffer in sequence. The shifts at *t* = 0 min correspond to a DOCP SAM. Summary of final (**C**) Δf and (**D**) ΔD shift values upon HLB formation relative to buffer baselines (prior to SAM formation).

We adopted the solvent exchange method from the SALB (solvent-assisted lipid bilayer) method [53] that includes the following steps: isopropanol (IPA) baseline, lipid adsorption in IPA, and lastly Tris buffer exchange. For these experiments, the DOPC lipid and Chol mixture were dissolved in IPA to 5 mg/mL and then diluted to 0.5 mg/mL before the lipid adsorption step, which occurred on the CP SAM-functionalized TiO_2_ surface. The kinetic profiles of the Δf and ΔD signals resembled those of SLB formation on TiO_2_ by the SALB method (Figure 3A,B) [54].

As reflected in the QCM-D kinetic traces, the exchange of Tris buffer solution to IPA in the measurement chamber initially resulted in transient signal spikes due to solvent mixing, followed by large, stable shifts reflecting the changes in bulk solution viscosity and density between Tris buffer and IPA. Relative to the IPA signal (prior to lipid addition), subsequent lipid addition (DOPC/Chol mixtures) in IPA to the measurement chamber led to much smaller shifts, indicating lipid adsorption at the SAM interface (as opposed to bulk solution effects). The final protocol step involved exchanging the bulk solution from IPA to Tris buffer, which again caused transient spikes in the measurement signals due to solvent mixing, followed by stable shifts that correspond to final upper leaflet formation. Relative to the initial QCM-D measurement signals in Tris buffer, the final QCM-D measurement signals were also obtained in Tris buffer, and thus the final reflected Δf and ΔD shifts could be attributed to lipid adsorption processes rather than bulk solution effects.

Compared to the initial baseline signals in neat Tris buffer (prior to SAM formation), the final Δf and ΔD values after Tris buffer exchange fell between −25 and −30 Hz and <1 × 10^−6^, respectively, indicating rigid HLB formation for all Chol fractions (Figure 3C,D). In particular, the Δf shifts of the DOPC/Chol upper leaflet were around −12 to −17 Hz depending on the Chol fraction, taking into account that the DOCP SAM itself corresponded to a Δf shift of around −13 Hz. Of note, the Δf shift tended to be larger at higher Chol fractions, and this trend is consistent with a previous report on DOPC/Chol SLBs formed using the SALB method [55]. In that study, the observed QCM-D results showed evidence of less dense membranes when the Chol fraction was below 20 mol%, in which range both the liquid disordered (L_d_) and liquid ordered (L_o_) states are known to coexist in DOPC/Chol membranes. However, when the Chol fraction exceeds 20 mol%, the L_o_ state is known to predominate. A corresponding decrease in the area per lipid occurs above 20 mol% Chol, which is related to a phase transition and causes formation of a denser membrane arrangement [56]. This effect, in turn, causes a larger Δf shift magnitude due to an increase in net membrane density. A similar trend is manifested here, suggesting that the upper leaflet undergoes densification at higher Chol fraction. Altogether, the results show that the solvent exchange method is capable of fabricating complete HLBs on hydrophobic DOCP SAMs when using DOPC/Chol mixtures with up to 65 mol% Chol fraction.

#### 3.2.2. Vesicle Fusion

**Figure 4:**
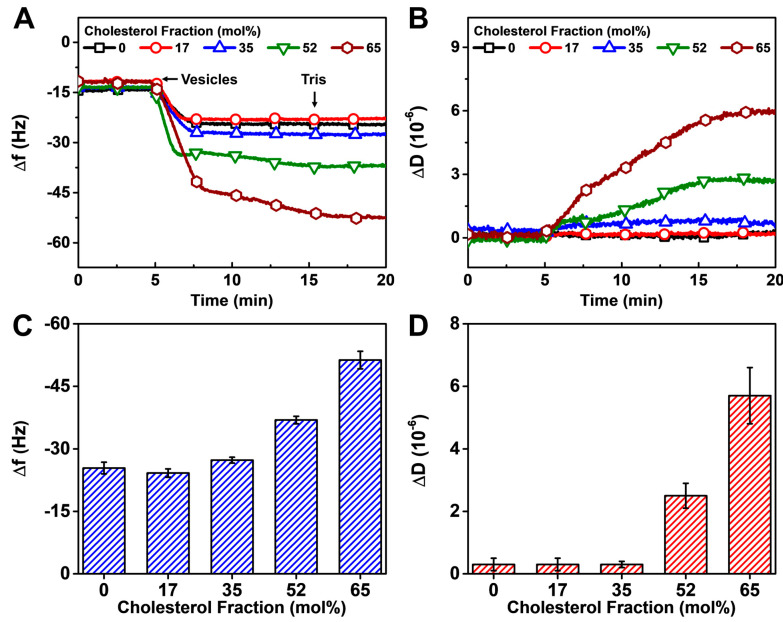
QCM-D monitoring of HLB formation with DOPC/Chol upper leaflets on DOCP SAMs by the vesicle fusion method. The QCM-D kinetic profiles of (**A**) resonance frequency (Δf) and (**B**) energy dissipation (ΔD) shifts during the upper leaflet formation process that includes DOPC/Chol vesicle addition and Tris buffer washing in sequence. The shifts at *t* = 0 min correspond to a DOCP SAM. Summary of final (**C**) Δf and (**D**) ΔD shift values upon HLB formation relative to buffer baselines (prior to SAM formation).

In the vesicle fusion method, DOPC/Chol lipid vesicles were added to DOCP SAMs, and, after a 10 min adsorption period, a Tris buffer washing step was subsequently performed to remove weakly adsorbed lipids. Vesicle adsorption with one-step kinetics occurred in all cases while close inspection of the final Δf and ΔD values enabled identification of the Chol fraction regime in which complete HLB fabrication occurred (Figure 4A,B). Indeed, the final Δf and ΔD values showed that upper leaflet formation was complete only in the cases of 0–35 mol% Chol, in which range the Δf shifts were around −25 to −30 Hz and ΔD shifts were <1 × 10^−6^, respectively, relative to original buffer baselines (prior to SAM formation) and indicated that rigid HLB formation occurred via complete vesicle rupture on the DOCP SAM (Figure 4C,D). The QCM-D Δf shift magnitude verified the expected range of adsorbed lipid mass, while the QCM-D ΔD shift magnitude established that the HLB behaved as a rigid adsorbate in a similar manner to other rigid lipid films like SLBs. By contrast, the final Δf shifts were around −37 and −51 Hz for the 52 and 65 mol% Chol cases, respectively, and the corresponding ΔD shifts were around 2.5 × 10^−6^ and 5.7 × 10^−6^, which point to ineffective HLB formation due to incomplete vesicle rupture on the DOCP SAM. The latter determination could be made by the combination of high Δf and ΔD signals that respectively reflect appreciable adsorbed lipid mass and the presence of extensive hydrodynamically coupled solvent beyond the levels that typically occur in rigid lipid films such as complete HLBs. This finding is consistent with the high bending rigidity of cholesterol-enriched vesicles at high Chol fractions, which can impede the rupture process [57,58]. Accordingly, the results support that the vesicle fusion method is capable of fabricating complete HLBs on DOCP SAMs when using DOPC/Chol mixtures with only up to 35 mol% Chol fraction.

Regarding stability of the fabricated HLBs, the QCM-D measurement shifts remained stable for several hours post-fabrication, which is consistent with the rugged character of HLBs due to the covalently attached SAM layer. While we did not perform long-term time stability studies beyond a few hours, a large body of literature supports the high stability of HLBs in general. In the context of Chol-enriched HLBs, we were particularly interested in ensuring sufficient buffer washing to remove weakly adsorbed phospholipids and/or sterols. This protocol point was particularly important when preparing HLBs from the 65 mol% Chol input fraction because this fraction is close to the Chol solubility limit in PC membranes [59]. The high measurement stability of HLB platforms, even in the high Chol case, was evidenced by negligible changes in the QCM-D signals upon extensive, flow-through washing in the measurement chambers.

### 3.3. Estimation of Chol Amount in HLB Upper Leaflet

We further estimated the amount of Chol in the upper leaflet of HLBs by extracting Chol with 1 mM MβCD treatment (which enables the specific removal of Chol from lipid membranes [60,61,62]) and calculating the mass loss based on Δf shifts according to the Sauerbrey equation [63]. At concentrations below 10 mM, MβCD exhibits a specific affinity for Chol and has been reported to neither interact with complete DOPC lipid membranes nor cause membrane-disruptive effects [60]. As such, MβCD has been widely used by our group and other groups [40,41,60,61,62] to extract Chol from supported lipid membranes in order to quantify the amount of Chol incorporated based on the corresponding QCM-D Δf shift values for the lipid membrane platform before and after MβCD treatment. Here, we focused on the upper leaflet because the close-packed DOCP SAM was fabricated prior to Chol introduction into the system and aimed to compare the input ratio of Chol (added to fabricate the HLB) vs. the actual ratio of Chol in the HLB upper leaflet by taking into account the DOPC and Chol molecular weights. Accordingly, the Chol fractions measured by the MβCD extraction assay can be correlated with the input amount of Chol in order to control the Chol fraction in the fabricated HLB platform as desired.

**Figure 5:**
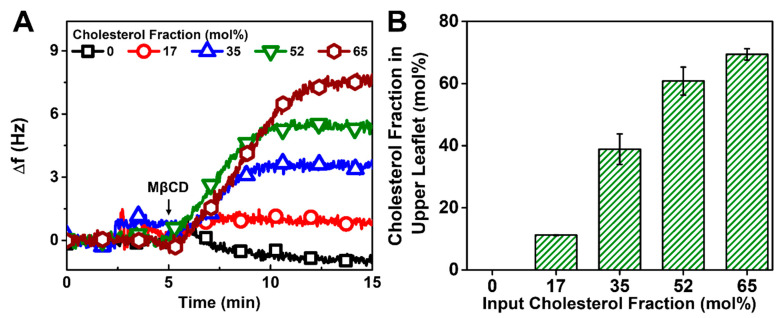
QCM-D monitoring of Chol extraction by MβCD from HLBs formed via solvent exchange. (**A**) Kinetic profiles of QCM-D frequency shifts (Δf) during the Chol extraction process. The zero Δf shifts at *t* = 0 min correspond to fabricated HLBs. (**B**) Calculated mol% Chol in the upper leaflet vs. mol% Chol in DOPC/Chol dispersion used to prepare the HLB.

In the case of the solvent exchange method, the addition of MβCD led to positive Δf shifts except for the 0 mol% Chol case, indicating mass loss due to the extraction of Chol molecules by MβCD (Figure 5A). The calculated Chol fraction in the upper leaflet was largely proportional to the input Chol fraction in each case (Figure 5B). However, in the case of ≥35 mol% Chol, the calculated mol% Chol in the HLB was around 10 mol% higher than the mol% Chol in the dispersed samples, which suggests preferential association of Chol molecules at the DOCP SAM interface. Overall, these results are consistent with the kinetic data that showed complete HLB formation in all cases (cf. Figure 3). Additionally, the data support that HLBs are uniform, as otherwise, the Δf shifts would have initially decreased upon the introduction of MβCD if there were any holes present in the HLBs.

**Figure 6:**
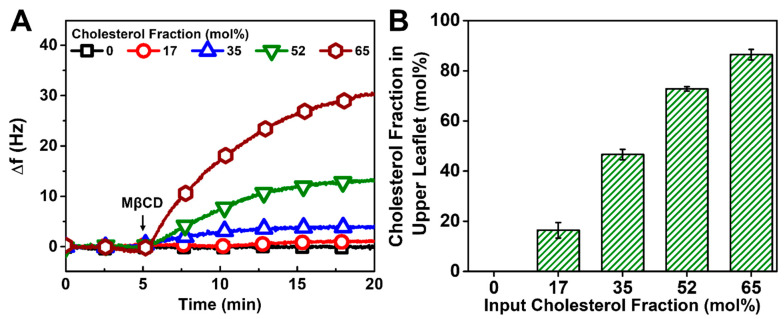
QCM-D monitoring of Chol extraction by MβCD from HLBs formed via vesicle fusion. (**A**) Kinetic profiles of QCM-D frequency shifts (Δf) during the Chol extraction process. The zero Δf shifts at *t* = 0 min correspond to fabricated HLBs. (**B**) Calculated mol% Chol in the upper leaflet vs. mol% Chol in DOPC/Chol vesicles used to prepare the HLB.

In the case of the vesicle fusion method, the addition of MβCD also led to positive Δf shifts, indicating mass loss due to Chol extraction (Figure 6A). Interestingly, the calculated Chol fraction in the upper leaflet was similar to the input Chol fraction only in the 17 mol% Chol case and was around 10% higher in the upper leaflet for the 35 mol% Chol case (Figure 6B). Both of these cases corresponded to complete HLB formation according to the kinetic data, and the latter result reinforces that Chol appears to preferentially adsorb at the DOCP SAM interface. Furthermore, the measured Chol fraction in the HLB upper leaflet was about 20 mol% higher than the input Chol fraction in the 52 and 65 mol% Chol cases. As discussed above, vesicle rupture is mediated by hydrophobic interactions upon interacting with the SAM and is efficient at lower Chol fractions [64]. On the other hand, based on the kinetic data, the vesicle rupture process is incomplete at higher Chol fractions so unruptured vesicles could contribute a larger amount of Chol molecules per HLB surface area, which would lead to the higher measured Chol fraction as an overestimate since the Sauerbrey equation would no longer be strictly valid [65,66]. Of note, there is typically a lower Chol amount in vesicles after extrusion than the input Chol amount when the precursor lipid mixture contains >33 mol% Chol [67], so the effect of unruptured vesicles is particularly noteworthy. Altogether, these results suggest that rigid HLBs formed only with 17 and 35 mol% Chol and agree with the kinetic data that showed incomplete HLB formation due to unruptured vesicles at >35 mol% Chol (cf. Figure 4).

### 3.4. Comparison of Chol Incorporation Effects by Different Fabrication Methods

The above findings confirm that the solvent exchange method can incorporate a higher Chol amount in the upper leaflet of HLBs on DOCP SAMs than the vesicle fusion method. This fabrication result can be rationalized by taking into account differences in the surface self-assembly behavior of the precursor lipid structures in each case. For the solvent exchange method, the precursor lipids were prepared by simply mixing the Chol molecules with DOPC lipids in IPA so, upon solvent exchange, the Chol distribution and movement are likely random during the phase transformations [68,69,70]. At high Chol fractions, some Chol molecules likely remain free in the bulk without being trapped in certain lipid structures even after phase transformations and may preferentially interact with the DOCP SAM interface. This effect leads to Chol enrichment in the HLB, as illustrated by the MβCD data (cf. Figure 5). Therefore, Chol incorporation is not limited and can be spontaneous, which likely explains the ability of the solvent exchange method to incorporate a high Chol amount in the upper leaflet. Moreover, the Chol incorporation capacity of HLBs is similar to that of SLBs formed on SiO_2_ [40,41], which demonstrates the usability of the solvent exchange method in forming Chol-enriched bilayers on both hydrophilic oxide surfaces and hydrophobic SAMs. This versatility is important to note because the self-assembly of phospholipid molecules on hydrophilic surfaces is mainly dictated by the balance of noncovalent interfacial forces such as electrostatic and van der Waals forces (and hence sensitive to environmental factors such as solution pH and ionic strength), whereas self-assembly of phospholipid molecules on hydrophobic surfaces is strongly influenced by hydrophobic interactions since the lower leaflet is already covalently attached to the surface and the main driving force is to form the upper leaflet.

By contrast, the precursor lipids for the vesicle fusion method were prepared in the form of extruded vesicles. As mentioned above, only when the Chol amount in the precursor lipids is less than ~33 mol% can the full Chol amount be incorporated into vesicles prepared by extrusion [67]. This range is consistent with the observed complete HLB formation that occurred when using precursor vesicles with 17 and 35 mol% Chol fractions (cf. Figure 4) and the corresponding Chol incorporation results showed good agreement (cf. Figure 6). For higher Chol fractions, the kinetic data (cf. Figure 4) and MβCD data (cf. Figure 6) conclusively showed incomplete vesicle rupture on the DOCP SAM, which led to unsuccessful HLB formation and thus overestimates of Chol fraction due to the presence of unruptured vesicles. Altogether, our results demonstrate that the vesicle fusion method can incorporate up to around 45 mol% Chol in HLBs that are formed on DOCP SAMs with precursor vesicles containing 35 mol% Chol. Hence, the Chol incorporation capacity of the vesicle fusion method is lower than that of the solvent exchange method to fabricate HLBs using DOCP SAMs. However, the capacity is still higher than that of the vesicle fusion method to fabricate SLBs on SiO_2_ under the same buffer conditions. In the latter case, only up to around 10 mol% Chol could be incorporated into SLBs using precursor vesicles containing 20 mol% Chol [40,41]. This difference supports that DOCP SAMs on TiO_2_ are useful interfaces upon which biomimetic, cholesterol-enriched lipid membranes can be prepared compared to other possible surface options like hydrophilic SiO_2_.

Regarding SAMs, thiol SAMs are commonly employed to fabricate HLBs on gold surfaces, while recent works have reported the use of one-tail silane SAMs to fabricate HLBs on Ti surfaces with an upper leaflet containing 40 mol% Chol via vesicle fusion [20,38,39]. Alongside the practical advantages of our CP-based SAM interface, including compatibility with aqueous conditions, reduced fabrication time, and operation at ambient temperature, we may also remark on reported properties of silane SAMs in general and how they compare to DOCP SAMs structure-wise. Past studies report a tendency for silane SAMs to exhibit a higher level of defects compared to thiol SAMs [39]. These defects can have an impact on the uniformity of upper leaflet coverage, consequently introducing certain levels of defects within the HLB structure. In contrast, the densely packed and homogeneous nature of the DOCP SAM in our study allows for achieving a uniform and complete coverage of the upper leaflet. In terms of fabrication ease and resulting SAM properties, it thus appears that CP-based SAM interfaces have attractive merits for HLB fabrication.

Looking forward, the findings in this study can be extended to other medically relevant inorganic surfaces such as Fe_3_O_4_ and ZrO_2_ [26] that are amenable to covalent bonding with phosphate-functionalized molecules such as DOCP. By studying the mechanisms of HLB formation on these other surfaces, it may be possible to engineer more effective and tailored interfaces for various biomedical applications. As demonstrated above, the solvent exchange method outperforms the vesicle fusion method in incorporating higher levels of Chol in HLBs. However, it is essential to recognize that the solvent exchange method requires the use of a water-miscible organic solvent for lipid deposition and thus also takes a longer time to conduct the protocol. Furthermore, when incorporating proteins into HLBs, the fully aqueous vesicle fusion method could be more advantageous, while its capacity for Chol incorporation is comparatively limited.

## 4. Conclusions

In this study, we have fabricated HLBs with Chol-containing upper leaflets via the solvent exchange and vesicle fusion methods on DOCP SAM-coated TiO_2_ surfaces. Our objective was to explore the feasibility of fabricating Chol-enriched HLBs on TiO_2_ using DOCP SAMs and to correlate the level of Chol incorporation in the HLB upper leaflet with the choice of fabrication method and input Chol fraction. We used the QCM-D characterization technique to evaluate HLB formation, and the results showed that, for complete HLB formation, precursor lipids with up to 65 mol% Chol can be used to form the upper leaflets via the solvent exchange method. By contrast, only up to 35 mol% Chol could be used with the vesicle fusion method. The structural difference between Chol molecules dispersed in organic solvents or organized within well-packed vesicles depending on the method, helps to rationalize the difference in Chol-incorporating capacity between the two methods. Furthermore, the tightly packed DOCP SAM likely contributes to the formation of homogeneous HLBs. While the QCM-D technique provided a versatile measurement tool to quantitatively characterize real-time lipid adsorption, SAM and HLB adlayer properties, and the level of Chol incorporation, future studies could also further investigate additional aspects like how Chol level affects mechanical strength. Altogether, the findings in this work demonstrate that the solvent exchange method facilitates a higher capacity than the vesicle fusion method for incorporating Chol molecules into HLB upper leaflets and can be useful for a wide range of surface biofunctionalization options on TiO_2_ as well as other medically important inorganic surfaces.

In closing, we also wish to briefly recap the benefits of using TiO_2_ surfaces for HLB fabrication from a biomimetic perspective. First, the possibility to use DOCP lipids to form two-tail SAMs on TiO_2_ surfaces enabled a high degree of biomimicry, especially when compared to conventional use of one-tail SAMs. Second, TiO_2_ is a well-recognized biocompatible material that is commonly employed in biomedical applications, such as implants [71,72], and lipid bilayer coatings have attracted attention for enhancing biofunctionalization possibilities. Consequently, the well-controlled fabrication of DOCP-based HLB platforms on TiO_2_ surfaces that incorporate biologically important molecules such as cholesterol as well as coordination with divalent ions [73] could make a significant application impact by providing stable biomimetic coatings with enhanced biocompatibility and functionality.

## Data Availability

The raw data required to reproduce these findings are available from the corresponding authors on reasonable request.
